# Chelidonine Induces Concurrent Elevation of pSer-STAT3 and Bcl-2 Levels in a Mitotic Subpopulation of Human T-Leukemia/Lymphoma Cells

**DOI:** 10.3390/ijms27031200

**Published:** 2026-01-25

**Authors:** Saraa Baddour, János Szöllősi, László Mátyus, György Vámosi, István Csomós, Andrea Bodnár

**Affiliations:** 1 Department of Biophysics and Cell Biology, Doctoral School of Molecular Medicine, Faculty of Medicine, Research Center for Molecular Medicine, University of Debrecen, Egyetem tér 1, H-4032 Debrecen, Hungary; saraa.baddour@med.unideb.hu (S.B.); szollo@med.unideb.hu (J.S.); lmatyus@med.unideb.hu (L.M.); vamosig@med.unideb.hu (G.V.); csomos.istvan@med.unideb.hu (I.C.); 2 HUN-REN-DE Cell Biology and Signaling Research Group, University of Debrecen, Egyetem tér 1, H-4032 Debrecen, Hungary

**Keywords:** signal transducer and activator of transcription 3 (STAT3) signaling, chelidonine, B-cell lymphoma 2 (Bcl-2), interleukin-2 (IL-2), flow cytometry, confocal microscopy, human T-cell leukemia/lymphoma

## Abstract

Signal transducer and activator of transcription 3 (STAT3) is a transcription factor that regulates a broad spectrum of genes with oncogenic potential, thereby serving as a critical driver of tumorigenesis. Canonical STAT3 function is mediated through tyrosine phosphorylation, which enables dimerization and transcriptional activation, whereas serine phosphorylation of STAT3 has a postulated role in fine-tuning canonical functions and contributes to non-canonical roles as well. One of the transcriptional targets of STAT3 is the anti-apoptotic B-cell lymphoma 2 (Bcl-2) protein, itself subject to phosphorylation-dependent regulation. In this study, we investigated the effect of chelidonine, an alkaloid component of *Chelidonium majus* L., on STAT3/Bcl-2 signaling in human T leukemia/lymphoma cells, reported to have numerous effects in common with microtubule-targeting agents (MTAs). Flow cytometry and confocal microscopy revealed that chelidonine transiently increased both serine-phosphorylated STAT3 (pSer-STAT3) and Bcl-2 levels in a distinct subpopulation of cells, with near-complete overlap between the affected cells. This effect appeared at least partially independent of interleukin-2 (IL-2) and was associated with the M-phase of the cell cycle, as indicated by enhanced phosphorylation of Bcl-2 at serine 70 and nuclear morphology characteristic of mitosis. Our study provides the first single-cell evidence that STAT3 and Bcl-2 undergo concurrent serine phosphorylation as a response to chelidonine treatment, with the effect tightly linked to the M-phase.

## 1. Introduction

Signal transducer and activator of transcription (STAT) proteins are a family of transcription factors that remain inactive in the cytoplasm until activated by extracellular signals. Among them, STAT3 plays a central role in disease progression, particularly in oncogenesis [1,2]. Activation of STAT3 is initiated by phosphorylation at the tyrosine 705 (Tyr705) residue, which promotes dimerization and subsequent nuclear translocation. Within the nucleus, STAT3 regulates the transcription of a broad spectrum of genes implicated in critical biological processes with oncogenic potential, including cell proliferation, apoptosis inhibition, and cell migration. STAT3 is activated by a wide range of cytokines and growth factors. Among these, interleukin-2 (IL-2), a key regulator of T cell function and homeostasis, can activate STAT3, despite STAT5 being the predominant downstream effector of IL-2 receptor signaling [3,4]. This activation is mediated mainly by janus kinase (Jak) 1 bound to the IL-2Rβ chain, which supports downstream STAT3 recruitment and activation [5,6].

In addition to Tyr705, STAT3 may also be phosphorylated at the serine 727 (Ser727) residue, a site targeted by various serine/threonine kinases such as mitogen-activated protein kinases (MAPKs), mammalian target of rapamycin (mTOR), and cyclin-dependent kinase 1 (CDK1) [1]. Serine phosphorylation of STAT3 is thought to be independent of tyrosine phosphorylation, yet it has been reported to contribute to the fine-tuning of STAT3 function [7,8,9]. Compelling evidence suggests that serine phosphorylation is essential for the optimal transcriptional activity of STAT3, exerting both positive and negative regulatory effects [9,10,11]. Serine phosphorylation has been proposed to contribute to the non-canonical functions of STAT3, including mitochondrial regulation and metabolic reprogramming, beyond its classical transcriptional role [12]. Elevated levels of serine-phosphorylated STAT3 (pSer-STAT3) were observed during mitosis, suggesting a potential role in the mitotic phase of the cell cycle [13].

One of the target gene products of STAT3 is the B-cell lymphoma 2 (Bcl-2) protein, an antiapoptotic member of the Bcl-2 protein family [14,15,16]. Regulation of Bcl-2 expression may also involve the participation of pSer-STAT3: in HeLa cells, phosphatidic acid induced Bcl-2 upregulation via activation of extracellular signal-regulated kinases 1 and 2 (ERK1/2), which in turn promoted STAT3 serine phosphorylation [17,18]. Notably, pSer-STAT3 binds to the *bcl-2* gene promoter even in the absence of tyrosine phosphorylation [18]. In addition to its role in controlling the intrinsic pathway of apoptosis by regulating mitochondrial membrane permeability, Bcl-2 also has non-canonical functions, including contribution to cell cycle control [19,20].

Bcl-2 contains multiple serine and threonine phosphorylation sites that play a crucial role in regulating the function of the protein [20,21,22]. Phosphorylation of Bcl-2, particularly at the serine 70 (Ser70) residue, is a physiological event during cell cycle progression, especially in the M-phase, and promotes cell survival [23,24,25]. However, under conditions of prolonged mitotic arrest induced by stress stimuli, Bcl-2 probably undergoes hyperphosphorylation, which impairs its anti-apoptotic function, thereby increasing cellular sensitivity for mitotic cell death [25,26]. Bcl-2 serine phosphorylation can occur independently of mitosis, influencing its stability and functional activity in response to cellular stress, oxidative damage, and oncogenic signaling [20,27,28].

Chelidonine, a benzophenanthridine alkaloid isolated from *Chelidonium majus* L., exhibits a broad spectrum of pharmacological activities, including antitumor, anti-inflammatory, and antimicrobial effects [29,30,31]. Chelidonine was reported to inhibit cell proliferation by inducing apoptosis and necrosis in various human cancer cell types [32,33,34]. Previous studies have reported that chelidonine shares mechanistic similarities with microtubule-targeting agents (MTA), many of which are clinically approved drugs used in cancer therapy [35,36]. In vitro studies suggested that chelidonine binds to or near the colchicine-binding site on the β-subunit of tubulin dimers, thereby disrupting microtubule dynamics, ultimately leading to cell cycle arrest at the G2/M phase [37,38,39]. In addition to inducing mitotic cell death through direct interference with the microtubular system, MTAs also act on other cellular targets, which may be consequences of their effects on microtubules or may occur independently [35,40]. Among others, several members of the MTA family were reported to modulate STAT3 signaling by affecting both the activation and serine phosphorylation of the transcription factor [41,42]. Previously, our research group obtained similar results with chelidonine in human uveal melanoma cells: chelidonine abrogated IL-6-induced activation and nuclear translocation of STAT3, but amplified serine phosphorylation of the transcription factor [32].

In the present study, we investigated whether chelidonine modulates STAT3 signaling in human T leukemia/lymphoma cells as well. Kit225 K6 cells are IL-2-dependent human T cells of mature T-cell leukemia/lymphoma origin, characterized by stable expression of all subunits of the heterotrimeric IL-2 receptor and serving as a useful model for IL-2/STAT signaling in activated T cells and T-cell malignancies, including T-cell lymphomas [5,43,44,45]. Additionally, we examined whether chelidonine affects the expression and phosphorylation status of Bcl-2 and explored potential correlations between its impact on STAT3- and Bcl-2-related cellular responses. To examine cellular responses at the single-cell level, we utilized flow cytometry and confocal microscopy. These methods allowed us to detect differences within the cell populations and to correlate various cellular responses with each other.

Flow cytometry analysis demonstrated that chelidonine increased both pSer-STAT3 and Bcl-2 levels in Kit225 K6 cells. These effects were confined to a distinct subpopulation of cells, occurring in an all-or-none manner, and showed considerable overlap, as further validated by confocal microscopy. The effect was transient and remained detectable—albeit at a reduced level—even in the absence of IL-2. Furthermore, it was associated with the M-phase of the cell cycle, supported by increased phosphorylation of Bcl-2 at the serine 70 residue and nuclear morphology characteristic of mitosis.

Taken together, our findings provide further evidence that the mechanism of action of chelidonine bears similarity to that of microtubule-targeting agents, suggesting that it may exert its effects through modulating mitotic processes. Since both STAT3 and Bcl-2 are considered potential targets in cancer therapy, our results raise the possibility of future therapeutic applications of chelidonine.

## 2. Results

### 2.1. Chelidonine Increases the Level of Serine-Phosphorylated STAT3 in a Distinct Subpopulation of Kit225 K6 Cells

To investigate the impact of chelidonine on serine phosphorylation of STAT3, Kit225 K6 human T leukemia/lymphoma cells were cultured in the presence of chelidonine or dimethyl sulfoxide (DMSO) alone (vehicle control) for different durations (6, 12, and 24 h). The level of pSer-STAT3 was assessed by flow cytometry, detecting the binding of anti-pSer-STAT3 monoclonal antibody (mAb). The two concentrations of chelidonine used in our experiments (0.25 and 0.5 μg/mL) were selected based on cell viability assays (Figure S1). Neither concentration induced significant cell death compared to the control following 6- or 12-h chelidonine exposure. However, after 24 h, the fraction of dead cells rose notably for the higher concentration compared to the control (~30% vs. 10%). Although statistically significant, the increase in cell death at the lower concentration was modest compared to the control (~14% vs. 10%).

**Figure 1:**
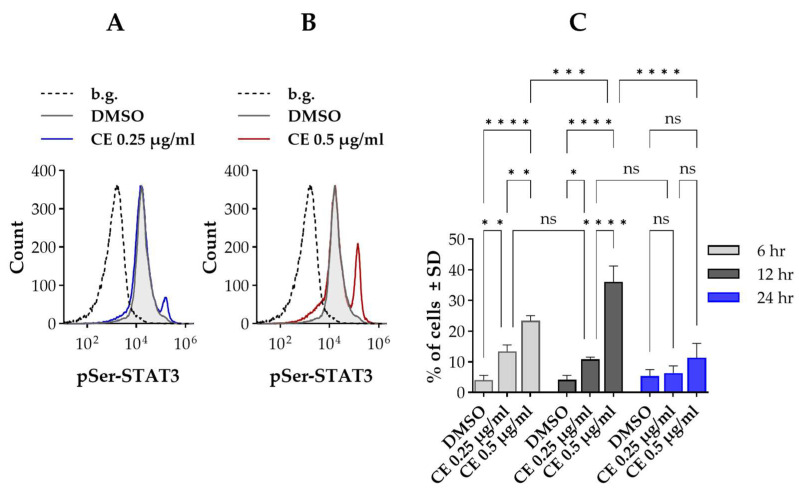
Chelidonine increases serine phosphorylation of signal transducer and activator of transcription 3 (STAT3) in a subpopulation of Kit225 K6 cells. (**A**,**B**) Representative flow cytometric histograms showing cell-by-cell distribution of pSer-STAT3, detected with Alexa Fluor 647-conjugated monoclonal antibody (mAb) specific for pSer727-STAT3, in cells treated with DMSO (vehicle control) or chelidonine. The gray-filled histogram denotes control cells, while blue and red histograms correspond to 0.25 and 0.5 μg/mL chelidonine treatments, respectively. The dashed histogram indicates the unstained background. (**C**) Bar chart showing the percentage of cells with elevated pSer-STAT3 levels (pSer-STAT3^HIGH^ cells) after 6-, 12- and 24-h treatment with chelidonine or DMSO alone (light gray, dark gray and blue bars, respectively). Percentages are expressed as the mean ± SD values of three independent experiments. * *p* < 0.05, ** *p* < 0.01, *** *p* < 0.001, **** *p* < 0.0001, ns: not significant. The percentage of pSer-STAT3^HIGH^ cells remained unchanged in DMSO-treated controls throughout the experimental time course. For clarity, statistical comparisons for control cells are not shown in the figure. Kit225 K6 cells were cultured with chelidonine or DMSO alone for the above-mentioned durations in the presence of 200 units/mL interleukin-2 (IL-2). Cells were then subjected to immunofluorescence staining and analyzed by flow cytometry (n = 100,000 cells/sample). (b.g.: background, CE: chelidonine, DMSO: dimethyl sulfoxide, pSer-STAT3: STAT3 phosphorylated on the serine 727 residue).

According to flow cytometric analysis (Figure 1), the serine-phosphorylated form of STAT3 could be detected in control Kit225 K6 cells, with a small subpopulation (~4–6%) characterized by elevated expression (referred to as pSer-STAT3^HIGH^ cells) compared to the rest of the cells (Figure 1C). Treatment with chelidonine led to an increase in the proportion of pSer-STAT3^HIGH^ cells at both tested concentrations and at all examined time points, relative to the control. Otherwise, the levels of pSer-STAT3 in the two subpopulations—reflected by the positions of their respective histograms—were comparable to those observed in the non-treated samples (Figure 1A,B).

The increase in the fraction of pSer-STAT3^HIGH^ cells could be observed even after the shortest duration tested (6 h). The effect was more prominent for the higher chelidonine concentration (0.5 µg/mL), but was evident for the lower concentration (0.25 µg/mL) as well (~23% vs. 13%, respectively). The fraction of pSer-STAT3^HIGH^ cells showed biphasic behavior with the duration of treatment: it increased up to ~36% after 12 h, followed by a decline to ~11% after 24 h for the higher dose of chelidonine. In the case of the lower concentration, there was no significant difference between the fraction of pSer-STAT3^HIGH^ cells for durations 6 and 12 h, but it declined to the level of control cells (~8%) after 24 h (Figure 1C). The fraction of pSer-STAT3^HIGH^ cells remained unaltered in the control sample throughout the time course of chelidonine treatment (Figure 1C).

### 2.2. Chelidonine Increases the Level of Bcl-2 in a Distinct Subpopulation of Kit225 K6 Cells

We further investigated the effect of chelidonine on the expression of the anti-apoptotic Bcl-2 protein, a STAT3 target gene product, under the same experimental conditions described in Section 2.1, employing immunofluorescence detection of Bcl-2.

**Figure 2:**
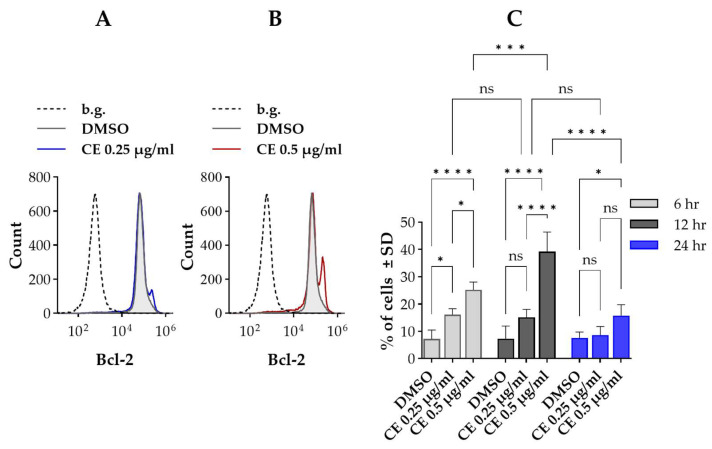
Chelidonine increases B-cell lymphoma 2 (Bcl-2) protein levels in a distinct subpopulation of Kit225 K6 cells. (**A**,**B**) Representative flow cytometric histograms showing cell-by-cell distribution of Bcl-2, detected with phycoerythrin (PE)-conjugated mAb specific for Bcl-2, in cells treated with DMSO (vehicle control) or chelidonine. The gray-filled histogram denotes control cells, while blue and red histograms correspond to 0.25 and 0.5 μg/mL chelidonine treatments, respectively. The dashed histogram indicates the background stained with PE-conjugated irrelevant antibody (isotype control). (**C**) Bar chart showing the percentage of cells with elevated Bcl-2 levels (Bcl-2^HIGH^ cells) after 6-, 12- and 24-h treatment with chelidonine or DMSO alone (light gray, dark gray and blue bars, respectively). Percentages are expressed as the mean ± SD values of three independent experiments. * *p* < 0.05, *** *p* < 0.001, **** *p* < 0.0001, ns: not significant. The percentage of Bcl-2^HIGH^ cells remained unchanged in DMSO-treated controls throughout the experimental time course. For clarity, statistical comparisons for control cells are not shown in the figure. Kit225 K6 cells were cultured with chelidonine or DMSO alone for the above-mentioned durations in the presence of 200 units/mL IL-2. Cells were then subjected to immunofluorescence staining and analyzed by flow cytometry (n = 100,000 cells/sample). (b.g.: background, CE: chelidonine, DMSO: dimethyl sulfoxide).

Our results revealed that the level of Bcl-2 followed a bimodal cell-by-cell distribution similar to that observed for pSer-STAT3 (Figure 2). A small proportion of cells (~8%) had a higher level of Bcl-2 (Bcl-2^HIGH^ cells) compared to the bulk population in the control sample. Chelidonine exerted an all-or-none effect on Bcl-2 expression, comparable to that seen with pSer-STAT3: without changing the positions of the respective histograms, chelidonine increased the proportion of Bcl-2^HIGH^ cells in a concentration-dependent manner (Figure 2A,B). The time-course of the effect was also similar to that observed for the pSer-STAT3^HIGH^ subset (Figure 2C). The percentage of Bcl-2^HIGH^ cells did not change significantly throughout the treatment for the control (Figure 2C).

### 2.3. The Effects of Chelidonine on pSer-STAT3 and Bcl-2 Levels Overlap with Each Other and Related to the M-Phase of the Cell Cycle

**Figure 3:**
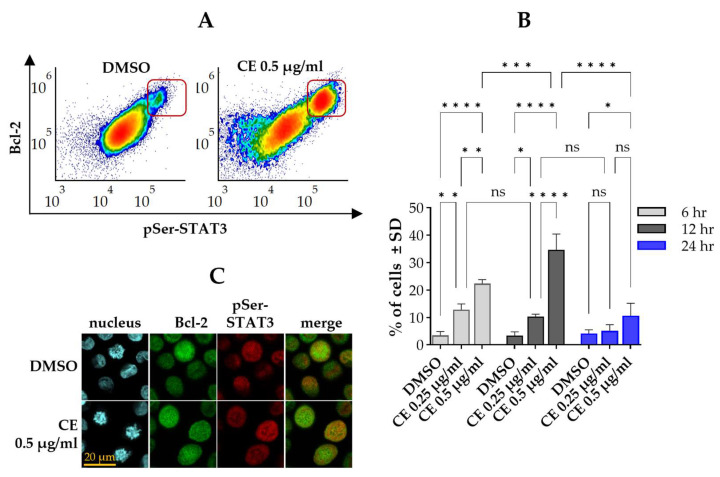
pSer-STAT3^HIGH^ and Bcl-2^HIGH^ subsets of Kit225 K6 cells show near-complete overlap. (**A**) Representative flow cytometric density dot plots showing pSer-STAT3 versus Bcl-2 levels in cells cultured with DMSO alone (left panel) or 0.5 μg/mL chelidonine (right panel) for 12 h. The framed subsets denote cells with elevated levels of both pSer-STAT3 and Bcl-2 (pSer-STAT3^HIGH^/Bcl-2^HIGH^ subset). Color reflects event density, with warmer colors representing regions containing more cells. (**B**) Bar chart showing the percentage of pSer-STAT3^HIGH^/Bcl-2^HIGH^ cells after 6-, 12- and 24-h treatment with chelidonine or DMSO alone (light gray, dark gray and blue bars, respectively). Data represent mean ± SD from three independent experiments. * *p* < 0.05, ** *p* < 0.01, *** *p* < 0.001, **** *p* < 0.0001, ns: not significant. The percentage of pSer-STAT3^HIGH^/Bcl-2^HIGH^ cells remained unchanged in DMSO-treated controls throughout the experimental time course. For clarity, statistical comparisons for control cells are not shown in the figure. (**C**) Representative confocal microscopy images showing coordinated changes in pSer-STAT3 and Bcl-2 levels in cells cultured with DMSO (top row) or 0.5 µg/mL chelidonine (bottom row) for 12 h. Blue fluorescence marks nuclei stained with 4′,6-diamidino-2-phenylindole (DAPI; first column), green fluorescence indicates Bcl-2 detected with phycoerythrin-conjugated anti-Bcl-2 (second column), and red fluorescence denotes pSer-STAT3 detected with Alexa Fluor 647-conjugated anti-pSer-STAT3 (third column). Overlay images (fourth column) show co-expression of pSer-STAT3 and Bcl-2. (Scale bar: 20 μm.) Kit225 K6 cells were cultured with chelidonine or DMSO alone for the above-mentioned durations in the presence of 200 units/mL IL-2. Cells were then subjected to immunofluorescence staining and analyzed by flow cytometry (n = 100,000 cells/sample) or confocal microscopy. (CE: chelidonine, DMSO: dimethyl sulfoxide, pSer-STAT3: STAT3 phosphorylated on the serine 727 residue).

Considering the similarities between the effects of chelidonine on the levels of pSer-STAT3 and Bcl-2 we wished to test whether there is a correlation between them. According to our data the pSer-STAT3^HIGH^ and Bcl-2^HIGH^ subpopulations almost completely overlapped with each other regardless the concentration or duration of chelidonine treatment and—as a consequence—followed the same biphasic time course discussed previously in Section 2.1 and Section 2.2 (Figure 3A,B).

Our findings were corroborated with confocal microscopy, proving that elevated levels of pSer-STAT3 and Bcl-2 are confined to the same subpopulation of cells (Figure 3C). Analysis of microscopic images also revealed that the affected cells are in the mitotic phase of the cell cycle. Further representative microscopic images are provided in the Supplementary Section (Figure S2).

### 2.4. Chelidonine Induces a Coordinated Elevation of Serine-Phosphorylated Bcl-2 and STAT3

**Figure 4:**
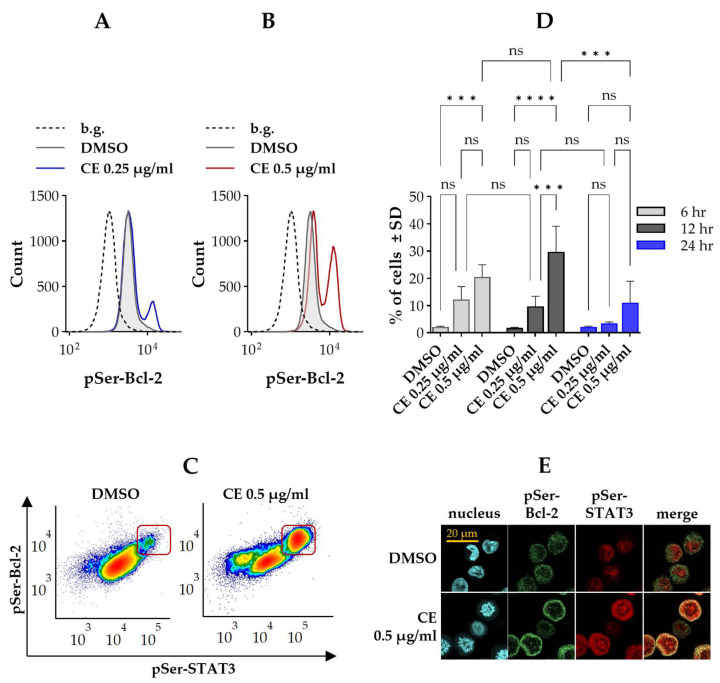
Chelidonine induces simultaneous and comparable increases in pSer-STAT3 and pSer-Bcl-2 levels within a subset of Kit225 K6 cells. (**A**,**B**) Representative flow cytometric histograms showing cell-by-cell distribution of pSer-Bcl-2, detected with Alexa Fluor 488-conjugated mAb specific for pSer70-Bcl-2, in cells treated with DMSO (vehicle control) or chelidonine. The gray-filled histogram denotes control cells, while blue and red histograms correspond to 0.25 and 0.5 μg/mL chelidonine treatments, respectively. The dashed histogram indicates the unstained background. (**C**) Representative flow cytometric density dot plots showing pSer-STAT3 versus pSer-Bcl-2 levels in cells cultured with DMSO alone (left panel) or 0.5 μg/mL chelidonine (right panel) for 12 h. The framed subsets denote cells with elevated levels of both pSer-STAT3 and pSer-Bcl-2 (pSer-STAT3^HIGH^/pSer-Bcl-2^HIGH^ subset). Color reflects event density, with warmer colors representing regions containing more cells. (**D**) Bar chart showing the percentage of pSer-STAT3^HIGH^/pSer-Bcl-2^HIGH^ cells after 6-, 12- and 24-h treatment with chelidonine or DMSO alone (light gray, dark gray and blue bars, respectively). Data represent mean ± SD from three independent experiments. *** *p* < 0.001, **** *p* < 0.0001, ns: not significant. The percentage of pSer-STAT3^HIGH^/pSer-Bcl-2^HIGH^ cells remained unchanged in DMSO-treated controls throughout the experimental time course. For clarity, statistical comparisons for control cells are not shown in the figure. (**E**) Representative confocal microscopy images showing coordinated changes in pSer-STAT3 and pSer-Bcl-2 levels in cells cultured with DMSO (top row) or 0.5 µg/mL chelidonine (bottom row) for 12 h. Blue fluorescence marks nuclei stained with DAPI (first column), green fluorescence indicates pSer-Bcl-2 detected with Alexa Fluor 488-conjugated anti-pSer-Bcl-2 (second column), and red fluorescence denotes pSer-STAT3 detected with Alexa Fluor 647-conjugated anti-pSer-STAT3 (third column). Overlay images (fourth column) show co-expression of pSer-STAT3 and pSer-Bcl-2. (Scale bar: 20 μm.) Kit225 K6 cells were cultured with chelidonine or DMSO alone for the above-mentioned durations in the presence of 200 units/mL IL-2. Cells were then subjected to immunofluorescence staining and analyzed by flow cytometry (n = 100,000 cells/sample) or confocal microscopy. (CE: chelidonine, DMSO: dimethyl sulfoxide, pSer-Bcl-2: Bcl-2 phosphorylated on the serine 70 residue, pSer-STAT3: STAT3 phosphorylated on the serine 727 residue).

Phosphorylation of Bcl-2 on the serine 70 residue (pSer-Bcl-2) was documented to occur during mitosis; therefore, we checked whether chelidonine has any effect on the phosphorylation status of Bcl-2. Flow cytometric analysis of cells doubly labeled with anti-pSer-STAT3 and anti-pSer-Bcl-2 mAbs revealed basal pSer-Bcl-2 in all cells, with a small fraction already showing elevated levels. Chelidonine treatment further increased the proportion of these pSer-Bcl-2^HIGH^ cells (Figure 4A,B). Moreover, the pSer-Bcl-2^HIGH^ subset exhibited a pronounced overlap with the pSer-STAT3^HIGH^ cells (Figure 4C). The fraction of cells with elevated levels of both pSer-STAT3 and pSer-Bcl-2 followed a similar biphasic time course as described previously for pSer-STAT3^HIGH^ and Bcl-2^HIGH^ cells (Figure 4D vs. Figure 1C, Figure 2C and Figure 3B).

Confocal microscopy analysis confirmed our findings: elevated levels of pSer-STAT3 and pSer-Bcl-2 were present on the same cells that are in the mitotic phase of the cell cycle (Figure 4E). The spatial distribution of pSer-Bcl-2 exhibited a distinct pattern relative to total Bcl-2: whereas Bcl-2 was more uniformly dispersed throughout the cell, pSer-Bcl-2 was predominantly concentrated in the perinuclear region (Figure 3C and Figure 4E). Further representative microscopic images are provided in the Supplementary Section (Figure S3).

### 2.5. Chelidonine-Induced Alterations in pSer-STAT3 and Bcl-2 Levels May Occur Independently of IL-2 Signaling

As a next step, we wished to determine whether the observed effects of chelidonine on pSer-STAT3 and Bcl-2 require the presence of IL-2. Since Kit225 K6 cells depend on IL-2 for their growth, prior to chelidonine treatment, cells were deprived of IL-2 and then treated with chelidonine (0.5 µg/mL) or DMSO alone for 12 h either in the presence or absence of the cytokine. The duration and concentration of the treatment were chosen based on our results presented in the previous sections, as this combination caused the highest effect among those tested. To get rid of the potential effect of IL-2 completely, cells were cultured in a medium supplemented with a reduced dose of IL-2 (30 units) and then, just prior to the experiment, were grown without IL-2 for 48 h (see Section 4).

**Figure 5:**
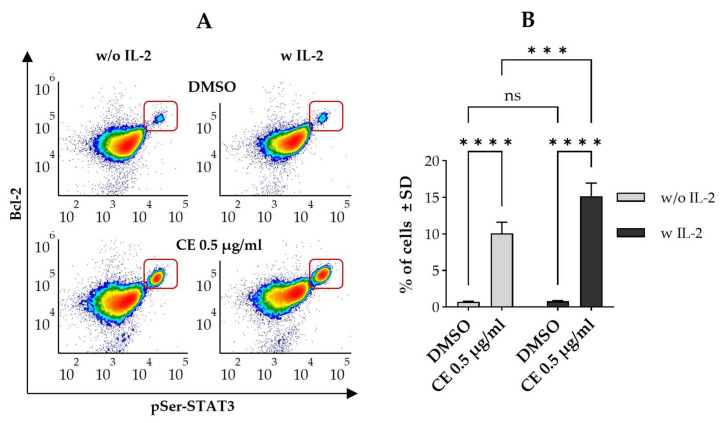
Chelidonine alters pSer-STAT3 and Bcl-2 levels independently of IL-2 signaling in Kit225 K6 cells. (**A**) Representative flow cytometric density dot plots showing pSer-STAT3 versus Bcl-2 levels in cells cultured with DMSO alone (top panels) or 0.5 μg/mL chelidonine (bottom panels) for 12 h, in the absence (left panels) or presence (right panels) of IL-2. The framed subsets denote cells with elevated levels of both pSer-STAT3 and Bcl-2 (pSer-STAT3^HIGH^/Bcl-2^HIGH^ subset). Color reflects event density, with warmer colors representing regions containing more cells. (**B**) Bar chart showing the percentage of pSer-STAT3^HIGH^/Bcl-2^HIGH^ cells treated with 0.5 μg/mL chelidonine in the presence (gray bars) or absence of IL-2 (black bars) for 12 h. Data represent mean ± SD from three independent experiments. *** *p* < 0.001, **** *p* < 0.0001, ns: not significant. Kit225 K6 cells deprived of IL-2 (see Section 4) were cultured with chelidonine or DMSO alone for 12 h in the absence or presence of 30 units/mL IL-2. Cells were then subjected to immunofluorescence staining and analyzed by flow cytometry (n = 100,000 cells/sample). (CE: chelidonine, DMSO: dimethyl sulfoxide, IL-2: interleukin-2, pSer-STAT3: STAT3 phosphorylated on the serine 727 residue, w and w/o: with and without).

Cells doubly labeled with anti-pSer-STAT3 and anti-Bcl-2 antibodies were analyzed by flow cytometry. Although it was lower than in our previous experiments, chelidonine significantly increased the percentage of pSer-STAT3^HIGH^/Bcl-2^HIGH^ cells compared to the control in the presence of IL-2 (~15% vs. 1%) (Figure 5). Lower, but still significant elevation could be observed for cells treated with chelidonine in the absence of IL-2 (~10% vs. 1%).

### 2.6. IL-2 Failed to Induce STAT3 Activation, and Chelidonine Likewise Showed No Significant Effect Under the Applied Experimental Conditions

As a next step, we wished to investigate whether chelidonine affects IL-2-induced activation of STAT3 in Kit225 K6 cells. In order to get rid of the potential effect of IL-2 present in the culture medium, cells were deprived of IL-2 as described in Section 2.5, treated with chelidonine (0.5 μg/mL, 12 h) or DMSO alone in the absence of IL-2, and then stimulated with IL-2 (200 units/mL, 30 min).

**Figure 6:**
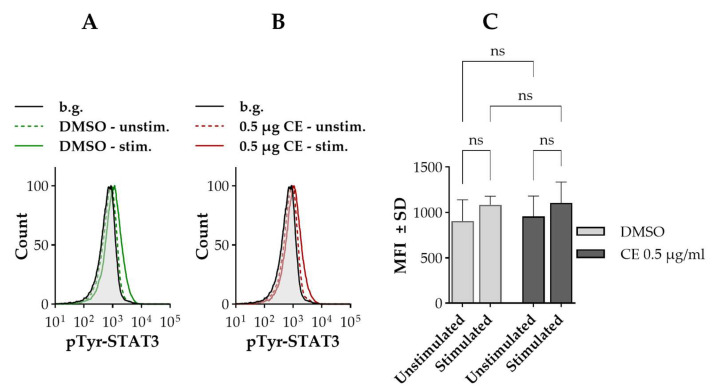
STAT3 tyrosine phosphorylation remains unaffected by IL-2 or chelidonine under the applied experimental conditions. (**A**,**B**) Representative flow cytometric histograms showing cell-by-cell distribution of pTyr-STAT3 in IL-2-stimulated and unstimulated cells treated with DMSO or chelidonine for 12 h. Green histograms represent DMSO-treated cells, while red histograms show chelidonine-treated cells: unstimulated (dashed) and IL-2-stimulated (solid). The gray-filled histogram indicates the unstained background. (**C**) Bar chart showing mean fluorescence intensity of pTyr-STAT3 distribution in cells cultured with DMSO (light gray bars) or 0.5 µg/mL chelidonine (dark gray bars), under unstimulated and IL-2-stimulated conditions. Data are expressed as the mean ± SD values of three independent experiments. ns: not significant. Kit225 K6 cells deprived of IL-2 (see Section 4) were cultured with 0.5 µg/mL chelidonine or DMSO alone for 12 h in the absence of IL-2, followed by stimulation with 200 U/mL IL-2 for 30 min. Cells were subsequently stained with Alexa Fluor 647-conjugated anti-pTyr-STAT3 mAb and analyzed by flow cytometry (n = 100,000 cells/sample). (CE: chelidonine, DMSO: dimethyl sulfoxide, IL-2: interleukin-2, MFI: mean fluorescence intensity, pTyr-STAT3: STAT3 phosphorylated on the tyrosine 705 residue).

According to our data, IL-2 stimulation failed to activate STAT3. Only a minor shift could be observed in the distribution of tyrosine-phosphorylated STAT3 (pTyr-STAT3) level, and the change was not statistically significant compared to unstimulated cells (Figure 6A,C). Furthermore, chelidonine did not have any effect on the observed activation pattern (Figure 6B,C).

**Figure 7:**
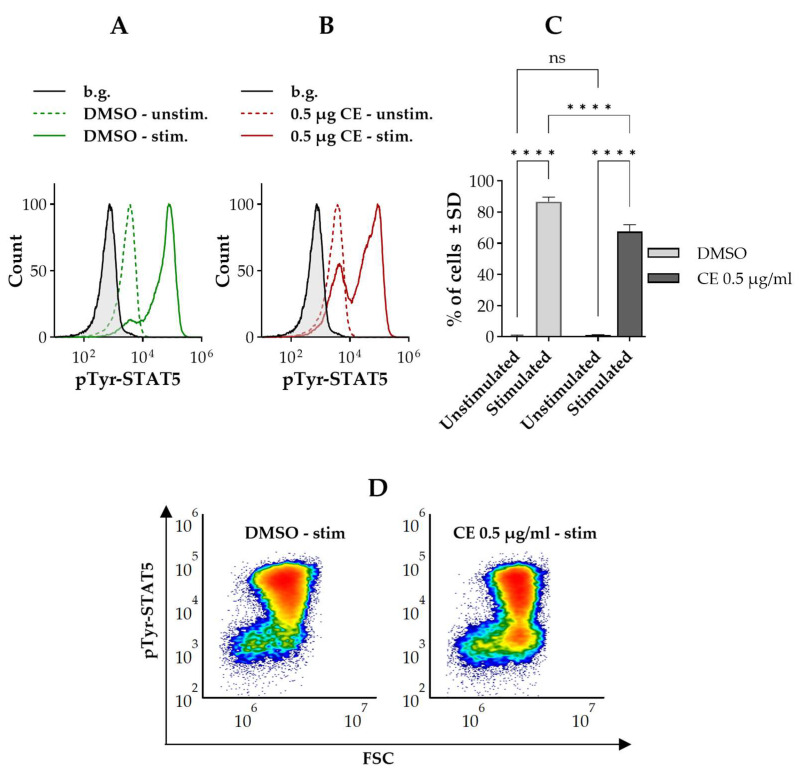
Chelidonine decreases IL-2-induced STAT5 activation in a subset of Kit225 K6 cells. (**A,B**) Representative flow cytometric histograms showing cell-by-cell distribution of pTyr-STAT5 in IL-2-stimulated and unstimulated cells treated with DMSO or chelidonine for 12 h. Green histograms represent DMSO-treated cells, while red histograms show chelidonine-treated cells: unstimulated (dashed) and IL-2-stimulated (solid). The gray-filled histogram indicates the unstained background. (**C**) Bar chart showing the percentages of cells with elevated levels of pTyr-STAT5 in cells cultured with DMSO (light gray bars) or 0.5 µg/mL chelidonine (dark gray bars), under unstimulated and IL-2-stimulated conditions. Data are expressed as the mean ± SD values of three independent experiments. **** *p* < 0.0001, ns: not significant. (**D**) Representative flow cytometric density dot plots showing pTyr-STAT5 versus forward scatter (FSC) in cells cultured with DMSO (left panel) or 0.5 µg/mL chelidonine (right panel) for 12 h, followed by IL-2 stimulation. Color reflects event density, with warmer colors representing regions containing more cells. Kit225 K6 cells deprived of IL-2 (see Section 4) were cultured with 0.5 µg/mL chelidonine or DMSO alone for 12 h in the absence of IL-2, followed by stimulation with 200 U/mL IL-2 for 30 min. Cells were subsequently stained with Alexa Fluor 647-conjugated anti-pTyr-STAT5 mAb and analyzed by flow cytometry (n = 100,000 cells/sample). (CE: chelidonine, DMSO: dimethyl sulfoxide, IL-2: interleukin-2, pTyr-STAT5: STAT5 phosphorylated on the tyrosine 694 residue).

In order to check whether the cells are still responsive to IL-2 under the applied experimental conditions, we examined whether IL-2 is capable of inducing tyrosine phosphorylation (activation) of STAT5, another—and in T cells generally more robust—downstream target of IL-2. According to flow cytometric analysis (Figure 7), IL-2 evoked activation of STAT5 in the vast majority of cells in the control sample (~85%) resulting in tyrosine phosphorylation of STAT5. Treatment with chelidonine significantly reduced the fraction of cells exhibiting STAT5 activation (~65%).

## 3. Discussion

Natural products, including alkaloids, are gaining growing interest for their potential to complement or replace conventional cancer therapy regimens [46]. Many of them, such as the vinca alkaloids, are already established in clinical use. Others, like berberine, an isoquinoline alkaloid, have advanced into clinical trials, for example, in colorectal cancer [47]. Chelidonine, a lesser-characterized isoquinoline alkaloid of the benzophenanthridine subtype, has also been reported to exhibit antitumor as well as diverse biological activities; however, its overall mechanism of action remains to be fully clarified [29].

Previously, we found that chelidonine induces cell death—via both apoptosis and necrosis—in uveal melanoma cells [32,33]. Our findings further revealed that chelidonine differentially modulates serine phosphorylation and IL-6–mediated activation of STAT3 [32]: it enhanced serine phosphorylation while suppressing activation and nuclear translocation of the transcription factor. In the present study, we demonstrated—using flow cytometry—that, beyond its cell-death-inducing effect (Figure S1), chelidonine similarly modulates serine-phosphorylated STAT3 levels in human T leukemia/lymphoma cells: it increased pSer-STAT3 in a distinct subset of cells in an all-or-none manner, and this effect appeared to be transient (Figure 1). At the higher chelidonine concentration applied (0.5 μg/mL), the fraction of responsive cells exhibited a biphasic pattern over the course of chelidonine treatment, with a peak observed at approximately 12 h based on analyses conducted at three time points: 6, 12, and 24 h. At the lower concentration applied (0.25 μg/mL), the transient nature of the response remained evident; however, the temporal profile differed, with the peak likely occurring earlier than 12 h.

Chelidonine also increased Bcl-2 expression, and the responsive cell populations showed near-complete overlap with the pSer-STAT3^HIGH^ subset, as revealed by both flow cytometry and confocal microscopy (Figure 2 and Figure 3). The Bcl-2^HIGH^ subset and the overlapping population followed the same temporal profile, similar to that of the pSer-STAT3^HIGH^ subset throughout the experiment, suggesting a functional link between these signaling events. Microscopic analysis revealed a prophase-like nuclear morphology in cells with elevated levels of pSer-STAT3 and Bcl-2 following chelidonine treatment, indicating that the effect of chelidonine on these two proteins is associated with the onset of M-phase (Figure 3). This observation is consistent with previous reports of pSer-STAT3 accumulation in mitotic cells under both physiological conditions and mitotic arrest induced by microtubule-targeting agents (MTAs) [13,41]. While Bcl-2 has been detected on mitotic chromosomes and is implicated in mitotic survival, direct evidence for its transcriptional upregulation during mitosis remains limited [48].

Chelidonine was reported to decrease Bcl-2 expression, affecting both mRNA and protein levels in B16F10 melanoma cells [49]. This observation contrasts with our findings, where Bcl-2 expression displayed a binary response to chelidonine: it was either stable or elevated, but never decreased (Figure 2). The discrepancy may reflect the use of a much higher chelidonine dose compared to the one we applied (2 μg/mL vs. 0.5 μg/mL, respectively), combined with bulk biochemical methods for assessing protein expression. Under such conditions, the strong cell-death-inducing effects likely bypassed G2/M arrest and masked heterogeneity in Bcl-2 regulation. This interpretation is further supported by their observation of cell cycle arrest in the G1 phase, in contrast to the G2/M arrest reported in our present and previous work and in several other studies (Figure S4) [32,37,38,39].

Our findings suggest that chelidonine may promote Bcl-2 expression in early mitosis. Bcl-2 expression—among other factors—is regulated by canonical STAT3 signaling. The dynamic change in pSer-STAT3 level may allow differential modulation of the transcriptional activity of STAT3. According to our data, chelidonine increased the levels of pSer-STAT3 and Bcl-2 in IL-2-depleted cells (i.e., cells cultured in IL-2-free medium), regardless of the presence of IL-2 during treatment (Figure 5). Although the proportion of affected cells was lower than in cells cultured with IL-2 (Figure 1, Figure 2 and Figure 3), the response remained detectable, even in the absence of IL-2 during chelidonine treatment, suggesting that the effect of chelidonine does not fully depend on IL-2. Moreover, the observed activity appears to be at least partially independent of tyrosine phosphorylation (activation) of STAT3, as tyrosine-phosphorylated STAT3 was virtually undetectable in IL-2-depleted cells, in contrast to those cultured in the presence of the cytokine (Figure S5). These findings suggest that chelidonine-induced upregulation of Bcl-2 is, at least in part, mediated by pathways independent of canonical STAT3 signaling. pSer-STAT3 was shown to bind the *bcl-2* promoter, potentially contributing to its transcriptional regulation, even in the absence of tyrosine phosphorylation of STAT3, thereby supporting a contribution from non-canonical STAT3 signaling [18]. It also cannot be excluded that the concomitant elevation of pSer-STAT3 and Bcl-2 represents parallel but independent consequences of the action of chelidonine on processes active during the early stages of M-phase.

Our previous and recent data consistently indicate that chelidonine affects STAT3 signaling by modulating processes regulating serine phosphorylation of the transcription factor. This effect is not cell-type-specific, as it was observed in cells of diverse origin in our present and previous work [32]. Together with the transient, all-or-none nature of the observed effect on pSer-STAT3 levels, these findings support our earlier hypothesis that at least two distinct cellular pools of pSer-STAT3 exist, likely regulated by different sets of kinases and other modulators [32]. Chelidonine selectively targets the pool associated with the onset of mitosis that is present under physiological conditions as well, as reflected by the minor fraction observed in control cells in our experiments (Figure 1). In addition, this pool can also be induced by various stress factors such as UV exposure, MTAs [13,42,50], or chelidonine, as demonstrated in both our previous and recent studies [32]. CDK1, the kinase driving mitotic entry, may regulate this pool. In serum-deprived HeLa cells released from G0/G1 arrest, pSer-STAT3 mirrored CDK1 activity, and in nocodazole-treated cells, it fluctuated similarly while total STAT3 remained stable [13]. In gastric cancer cells, chelidonine transiently increased cyclin B, the CDK1 activator, before its decline [34].

The similar pattern of Bcl-2 expression changes supports the existence of two regulatory pools for Bcl-2 as well: one constitutive and one inducible, likely responsive to mitosis-associated signaling, with chelidonine acting on the latter. Activated (tyrosine phosphorylated) STAT5 was reported to promote transcription of Bcl-2 in hematopoietic cells [51,52,53,54,55]. In contrast to STAT3, STAT5 remained constitutively active in our cells even under IL-2-depleted conditions (Figure 7 vs. Figure 6 and Figure S5). Given that Bcl-2 expression was already detectable prior to chelidonine treatment (Figure 2), it is plausible that this constitutive STAT5 activity sustains basal Bcl-2 transcription independently of cytokine exposure. Chelidonine may further enhance Bcl-2 expression through mitosis-associated signaling or non-canonical STAT3 activation.

Chelidonine not only altered Bcl-2 expression levels in parallel with pSer-STAT3, but also affected its phosphorylation state. The fraction of cells with elevated pSer-Bcl-2 (Bcl-2 phosphorylated at serine 70), which has been linked to mitotic processes under specific stress conditions, exhibited a simultaneous change with pSer-STAT3 and—given the near-complete overlap between pSer-STAT3^HIGH^ and Bcl-2^HIGH^ cells—with total Bcl-2 (Figure 4). Changes in pSer-Bcl-2 are in line with the proposed impact of chelidonine on mechanisms leading to elevated pSer-STAT3 levels associated with the onset of mitosis. These findings support the involvement of the CDK1 axis as a potential target of chelidonine, as CDK1 is known to phosphorylate not only STAT3, but also Bcl-2 in response to microtubule-targeting agents (MTAs) [13,56,57]. Further experiments will be required to clarify the involvement of CDK1. Approaches such as the use of selective CDK1 inhibitors or phospho-specific antibodies recognizing regulatory CDK1 epitopes may help determine, at the single-cell level, whether CDK1 contributes to the observed expression pattern of pSer-STAT3 or pSer-Bcl-2. Bulk CDK1 activity measurements could also provide complementary information.

The transient effect of chelidonine on pSer-STAT3 and Bcl-2/pSer-Bcl-2 raises the question of whether the subsequent decline in responsive cells reflects cell death. The increase in cell death between the 12-h and 24-h time points was (~16%) lower than the corresponding decrease in pSer-STAT3^HIGH^/Bcl-2^HIGH^ cells (~24%), indicating that the decline cannot be explained solely by cell death (Figure 1, Figure 2, Figure 3 and Figure 4 and Figure S1). In the vast majority of previously responsive cells, pSer-STAT3 levels reverted to the lower-expression state, rather than dropping below it, suggesting active regulation of phosphorylation rather than passive loss behind the transient nature of the effect. This implies that chelidonine promotes serine phosphorylation pathways without suppressing negative regulators, and that the signaling machinery remains functional. Moreover, the proportion of cells arrested in G2/M substantially exceeded the fraction of pSer-STAT3^HIGH^/Bcl-2^HIGH^ cells, indicating that the effect of chelidonine is not sustained throughout the mitotic block (Figure S4). Previously, we obtained similar results in uveal melanoma cells [32]. By 24 h, the marked reduction in G2/M-phase cells aligns with the onset of cell death at a later stage. In parallel, a subset of cells with pSer-STAT3 levels falling below the lower-expression state was observed in some experiments (e.g., Figure 4). These cells may represent non-viable populations in which the mechanisms sustaining basal pSer-STAT3 phosphorylation are no longer functional.

Regardless of whether changes in pSer-STAT3 and Bcl-2/pSer-Bcl-2 levels are causally linked or represent parallel outcomes of mitosis-associated processes, their elevation may contribute to enhanced cellular protection during mitosis, at least in the short term. Serine phosphorylation of STAT3 with mitochondrial localization was indicated to support viability under stress conditions, including mitosis [58,59,60]. In addition, the translocation and DNA binding of pSer-STAT3 may contribute to cell survival, as suggested in CLL cells [8]. Similarly, moderate-level phosphorylation of Bcl-2 at Ser70 enhances its antiapoptotic function [23,24]. In the cell line we examined, these proteins are constitutively present, with a small fraction of cells already showing elevated levels under control conditions that may contribute to coping with mitotic stress (Figure 1 and Figure 4). Chelidonine increases this proportion, triggering a transient rise that supports survival during early mitotic stress. However, this temporary signal diminishes during prolonged cell cycle arrest, eventually leaving the cells unable to prevent mitotic or post-mitotic death.

It should be noted that prolonged mitotic arrest can lead to Bcl-2 hyperphosphorylation (Ser70, Ser86 and Thr69) as reported under exposure to MTAs, including paclitaxel, vincristine, and nocodazole [21]. Bcl-2 hyperphosphorylation can diminish its protective role by altering conformation or reducing binding to proapoptotic partners such as Bax, and may even actively promote cell death [61]. However, in our system the transient nature of Ser70 phosphorylation suggests that sustained hyperphosphorylation due to chelidonine-induced mitotic arrest is unlikely.

Previously, we demonstrated that chelidonine abrogates IL-6-induced STAT3 activation in uveal melanoma cells, with partial overlap with the subset of cells exhibiting elevated pSer-STAT3 levels [32]. To assess whether this effect—like the modulation of pSer-STAT3—is also present in a distinct cellular context, we examined the impact of chelidonine on IL-2-induced STAT3 activation in Kit225 K6 cells. Given the IL-2-dependency of these cells, STAT3 activation assays were performed under IL-2-depleted conditions, with IL-2 excluded throughout chelidonine treatment to eliminate confounding effects. Under these conditions, IL-2 failed to activate STAT3, and chelidonine had no impact on STAT3 phosphorylation (Figure 6). Importantly, the cells remained responsive to IL-2, as STAT5 activation was still detectable in the majority of cells. However, chelidonine significantly increased the fraction of cells unresponsive to IL-2 stimulation. Based on our preliminary data, we propose that cell death alone does not account for this effect, as the pTyr-STAT5 level in nonresponsive cells did not fall below the constitutive activation level observed in non-stimulated control cells (Figure 7). Moreover, the forward scatter (FSc) signal remained preserved in the majority of these nonresponsive cells, further supporting their viability. The failure to activate STAT3 under these experimental conditions may reflect a complete shift in IL-2 signaling toward STAT5 activation. Indeed, STAT3 is not the primary downstream target of IL-2; rather, STAT5 is the dominant effector [3]. However, IL-2-induced STAT3 activation has also been observed, as in the case of the cell line used in our experiments [5]. This is supported by our previous findings [44], as well as by the pTyr-STAT3 levels detected in cells cultured in the presence of IL-2 in the present study (Figure S5).

In conclusion, our study shows that chelidonine-induced elevation in pSer-STAT3 level is likely cell-type-independent, as consistent results in T cells mirror those observed in uveal melanoma cells [32]. Using single-cell approaches such as flow cytometry and confocal microscopy, we revealed simultaneous changes in pSer-STAT3 and Bcl-2/pSer70-Bcl-2 levels linked to M-phase. Earlier studies examined these phosphorylation events separately with bulk assays that mask cell-by-cell heterogeneity, whereas the methods we applied provide direct evidence that they occur in parallel within individual mitotic cells. To our knowledge, this is the first demonstration at single-cell resolution that STAT3 and Bcl-2 phosphorylation dynamics coincide with each other and the M-phase.

Our findings on chelidonine reinforce its mechanistic similarity to microtubule-targeting agents and, considering its ability to overcome multidrug resistance [62], underscore this alkaloid as a promising candidate for combinational or multimodal cancer therapies. Targeting STAT3 and Bcl-2 is a promising approach in such strategies [63,64]. Emerging evidence indicates that, in addition to the tyrosine-phosphorylated form, pSer-STAT3 may also represent a relevant drug target in cancer treatment, given its recently uncovered role in cancer progression [1]. Elevated pSer-STAT3 and Bcl-2/pSer70-Bcl-2 during mitosis may define a dual therapeutic approach that can be employed by mitosis-focused strategies to disrupt cell division and overcome treatment resistance.

Although the present study was performed in leukemia/lymphoma cells, the transient STAT3- and Bcl-2–related signaling changes observed here suggest that similar early events may also be relevant in proliferating immune cells—particularly T lymphocytes—within the microenvironment of solid tumors, consistent with the indirect immune-modulatory effects described for MTAs [65]. Further investigation will be required to clarify whether these early signaling dynamics influence the function of proliferating immune cells in vivo.

## 4. Materials and Methods

### 4.1. Cell Line and Cell Culture

The Kit225 K6 cell line is a subclone of the IL-2–dependent human T-leukemia line Kit225 [66], originally derived from adult T-cell prolymphocytic leukemia and exhibiting a helper/inducer (CD4^+^) phenotype (kindly provided by Thomas A. Waldmann^†^, National Institute of Health, Bethesda, MD, USA; RRID: CVCL_5445). These cells do not express human T lymphotropic virus (HTLV), are cytokine-dependent and constitutively express all the subunits of IL-2R [43,44,66].

Cells were cultured in the presence of 200 units/mL IL-2 (Ro 23-6019, Hoffmann-La Roche, Basel, Switzerland; kindly provided by Thomas A. Waldmann^†^, National Institute of Health, Bethesda, MD, USA) in RPMI 1640 medium (R6504, Sigma-Aldrich, St. Louis, MO, USA) supplemented with 10% fetal bovine serum (FBS, F9665, Sigma-Aldrich, St. Louis, MO, USA), 2 mM L-glutamine (G3126, Sigma-Aldrich, St. Louis, MO, USA) and 10 units/mL penicillin and 100 µg/mL streptomycin (P4433, Sigma-Aldrich, St. Louis, MO, USA). The cells were incubated at 37 °C, in a humidified 5% CO_2_ atmosphere. The cells were subcultured three times per week.

For certain experiments, cells were subjected to IL-2 deprivation. Initially, they were cultured in medium containing a reduced concentration of IL-2 (30 units/mL). Cells were maintained under these conditions for up to a maximum of three weeks. Subculturing was performed using the same protocol as described above. Freshly harvested cells were then washed and transferred to IL-2–free medium for 48 h, after which they were designated as IL-2–deprived cells. For stimulation assays, these IL-2–deprived cells were treated with IL-2 at a final concentration of 200 units/mL for 30 min at 37 °C.

### 4.2. Chelidonine Treatment

After pelleting the cells (5 min, 200× *g*, 37 °C), the supernatant was removed and fresh medium was added to obtain the appropriate cell concentration (6 × 10^5^ cells/mL). 2 mL of the suspension was added to each well in a 6-well plate.

Chelidonine stock (54274, Sigma-Aldrich, St. Louis, MO, USA) was dissolved in dimethyl sulfoxide (DMSO; D2650, Sigma-Aldrich, St. Louis, MO, USA) at a concentration of 12 μg/μL. Before chelidonine treatment, the stock solution was serially diluted with DMSO so that the final concentration of DMSO (0.5 µL/mL) was the same in all related samples. Control cells were cultured in the presence of the same concentration of DMSO. The cells were cultured for different durations (6, 12 and 24 h) depending on the requirement of each experiment. At the end of the incubation periods, the cells were washed and then processed according to the specific requirements outlined for each experiment, as detailed later.

In experiments involving IL-2–deprived cells, treatment with chelidonine was carried out either in IL-2–free medium or in a medium supplemented with IL-2 at a concentration of 30 units/mL.

### 4.3. Annexin V and Propidium Iodide Assay

To evaluate the cell death-inducing effects of chelidonine, we applied combined Annexin V/Propidium Iodide (PI) staining. After chelidonine treatment, the cells were resuspended in Annexin V binding buffer (10 mM HEPES/NaOH, 0.14 M NaCl, 2.5 mM CaCl_2_, pH 7.5) at a concentration of 3 × 10^5^ cells/100 μL. The cells were incubated with 2.4 μg/mL of Annexin V-Alexa Fluor 647 (640912, BD Biolegend, San Diego, CA, USA) and 0.5 μg/mL PI (P4170, Sigma-Aldrich, St. Louis, MO, USA), and kept in the dark for 15 min at room temperature. The fluorescence of the samples was detected using flow cytometry right after the staining. Apoptotic and necrotic cells were identified based on Annexin V binding and PI exclusion.

### 4.4. Cell Cycle Analysis

To study the impact of chelidonine on cell cycle progression, we performed ethanol-fixed PI staining. Cells were harvested (~1 × 10^6^ cells per sample), and the cell suspension was pelleted (5 min, 200× *g*, 4 °C). The pellet was washed with 1 mL ice-cold PBS. 70% ice-cold ethanol was slowly added to prevent cell clumping (500 µL in two steps). Then cells were incubated at +4 °C for 1 h, then pelleted (5 min, 200× *g*, 4 °C) and washed twice with 1 mL PBS to remove ethanol completely. Cells were incubated with 100 µg/mL RNase A (10109142001, Merck, Darmstadt, Germany) at 37 °C for 15 min in the dark to degrade RNA, preventing background staining. Then cells were stained with 200 µL PI (50 µg/mL in PBS), incubated for 25 min at RT, washed with 1 mL PBS, and resuspended in 200 µL PBS. Proportions of cells in the various phases of the cell cycle (G0/G1, S, and G2/M) were determined based on the fluorescence intensity upon DNA-PI binding, which corresponds to the DNA content.

### 4.5. Immunofluorescence Labelling

Immunofluorescence staining was performed using the following antibodies, phycoerythrin (PE)-conjugated anti-human Bcl-2 (mouse IgG1, 556535, BD Pharmingen™, San Diego, CA, USA), Alexa Fluor 647-conjugated anti-human phospho-Ser727-STAT3 (mouse IgG1, 558099, BD Phosflow™, Franklin Lakes, NJ, USA), Alexa Fluor 647-conjugated anti human phospho-Tyr705-STAT3 (mouse IgG2κ, 558557, BD Phosflow™, Franklin Lakes, NJ, USA), Alexa Fluor 647-conjugated anti human phospho-Tyr694-STAT5 (mouse IgG1κ, 612599, BD Phosflow™, Franklin Lakes, NJ, USA), and Alexa Fluor 488-conjugated anti-human phospho-Ser70-Bcl-2 (mouse IgG1, 562678, BD Phosflow™, Franklin Lakes, NJ, USA) mAbs.

Cells were harvested and washed with PBS buffer (5 min, 200× *g*, 37 °C). After being fixed with formaldehyde (FA 2%) for 15 min at 37 °C, cells were washed with staining buffer (PBS + 2% fetal bovine serum + 0.1% sodium azide) and permeabilized in methanol 90% for 30 min at −20 °C, and washed twice in the staining buffer before being incubated with the appropriate antibody/antibodies (3 × 10^5^ cells/sample). The samples were incubated for 45 min at room temperature. Then cells were washed in the staining buffer, resuspended in FA 2% (400 µL), and measured by flow cytometry. Measurements were performed in triplicate for each treatment under identical conditions.

Nonspecific binding of monoclonal antibodies was checked using irrelevant antibodies having the same isotype. Since the differences between the fluorescence intensity histograms of the isotype controls and the unstained cells were negligible, the unstained samples were used as background in flow cytometry experiments. In the case of PE-Bcl-2, the signal from cells labeled with the PE-conjugated isotype control was higher than the background; however, the specific signal was still significantly higher, and thus remained distinguishable.

### 4.6. Flow Cytometry

Cell viability, cell cycle, protein expression, and phosphorylation were measured using NovoCyte 3000 RYB flow cytometer (Agilent, Santa Clara, CA, USA). The blue laser (488 nm) was used to excite propidium iodide and Alexa Fluor 488. Yellow (561 nm) and red lasers (640 nm) were used for phycoerythrin and Alexa Fluor 647, respectively. Their emissions were detected through 615/20, 530/30, 586/20, and 660/20 nm bandpass filters, consecutively. The measurements involved the detection of forward and side scatter (FSc and SSc) signals as well. The data analysis was performed using the FCS Express (version 6; De Novo Software, Pasadena, CA, USA).

### 4.7. Confocal Microscopy

Nikon A1 confocal microscope (Nikon Corporation, Tokyo, Japan) was used to visualize spatial distribution/co-distribution of fluorescently labeled Bcl-2, pSer70-Bcl2, and pSer-STAT3 within the cells. We also studied the changes in nuclear morphology in response to the different chelidonine treatments by staining the nuclei with 4′,6-diamidino-2-phenylindole (DAPI, 0.5 µg/mL, 62248, Thermo Fisher Scientific, Waltham, MA, USA).

Diode lasers with specific wavelengths (405 nm, 480 nm, 561 nm, and 647 nm) were used to excite the DAPI, Alexa Fluor 488, PE, and Alexa Fluor 647 dyes, and their fluorescence emissions were detected via 452/45, 525/50, and 593/46 nm bandpass filters, respectively.

Images of approximately 1-µm-thick optical sections, each containing 512 × 512 pixels, were captured with a Plan Apo IR 60X WI DIC N2 water immersion objective and were processed using the ImageJ software (version 1.54p) [67]. Images were taken in sequential mode to reduce crosstalk between the channels.

### 4.8. Statistical Analysis

Results are expressed as means ± SD. Statistical significance of differences between groups was assessed using Two-way ANOVA with post hoc Tukey. Curves were plotted using GraphPad Prism (version 9, GraphPad Software, San Diego, CA, USA). A *p*-value of less than 0.05 was considered statistically significant.

## Data Availability

The original contributions presented in this study are included in the article/Supplementary Material. Further inquiries can be directed to the corresponding author.

## Supplementary Materials

The following supporting information can be downloaded at: https://www.mdpi.com/article/10.3390/ijms27031200/s1.

## Abbreviations

The following abbreviations are used in this manuscript:

Bcl-2	B-cell lymphoma 2
CE	Chelidonine
CDK1	Cyclin-dependent Kinase 1
DAPI	4′,6-diamidino-2-phenylindole
DMSO	Dimethyl sulfoxide
Fsc	Forward scatter
IL-2	Interleukin-2
mAb	Monoclonal antibody
MFI	Mean Fluorescence Intensity
MTA	Microtubule Targeting Agent
PBS	Phosphate-buffered Saline
PE	Phycoerythrin
PI	Propidium iodide
pSer-Bcl-2	Bcl-2 phosphorylated on serine 70
pSer-STAT3	STAT3 phosphorylated on serine 727
pTyr-STAT3	STAT3 phosphorylated on tyrosine 705
pTyr-STAT5	STAT5 phosphorylated on tyrosine 694
STAT3 or 5	Signal transducer and activator of transcription 3 or 5
