# Supplementary material for: Chelidonine Induces Concurrent Elevation of pSer-STAT3 and Bcl-2 Levels in a Mitotic Subpopulation of Human T-Leukemia/Lymphoma Cells
Source: Int J Mol Sci. 2026 Jan 25;27(3):1200. doi: 10.3390/ijms27031200 (PMC12898084; doi:10.3390/ijms27031200)
Supplement: Supplementary file 1 [file ijms-27-01200-s001.zip › ijms-4080994-supplementary.pdf]

SUPPLEMENTARY FIGURES

Chelidonine Induces Concurrent Elevation of pSer-STAT3 and Bcl-2 Levels in a Mitotic Subpopulation of Human T-Leukemia/Lymphoma Cells

Saraa Baddour, János Szöllősi, László Mátyus, György Vámosi, István Csomós, Andrea Bodnár

SUPPLEMENTARY FIGURES

Supplementary Figure 1.

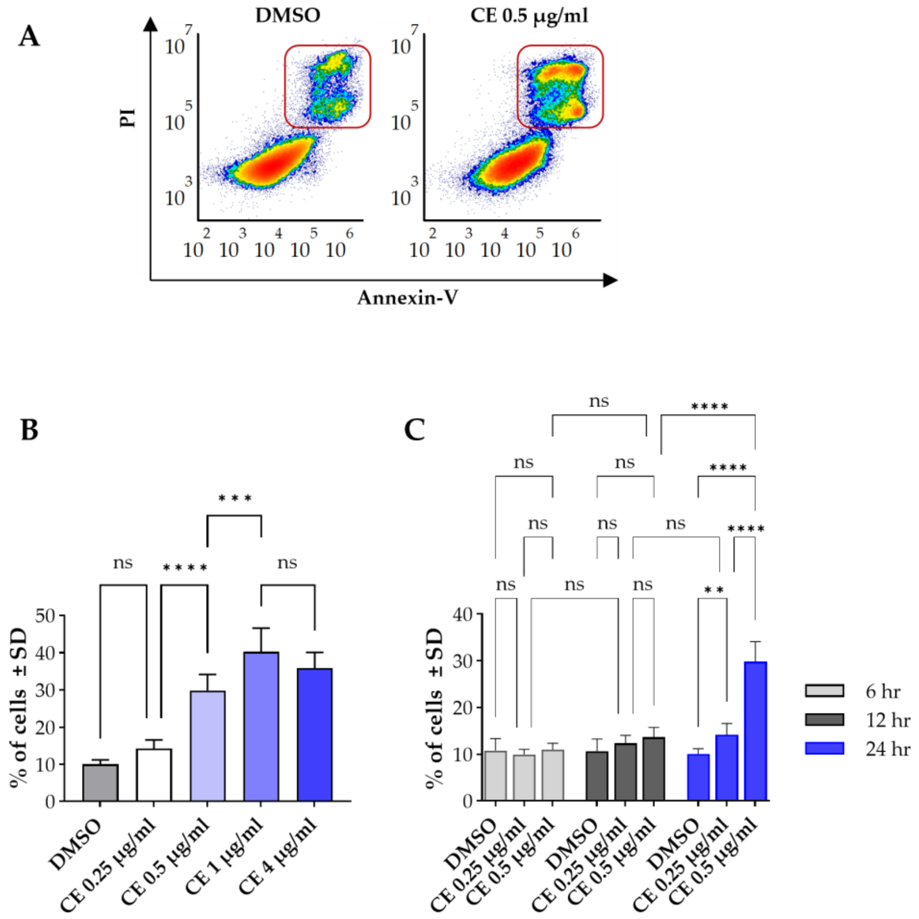

Chelidonine induces both apoptotic and necrotic cell death in Kit225 K6 cells, as determined by Annexin V/PI staining. (A) Representative flow cytometric density dot plots showing propidium iodide (PI) versus Annexin V staining in cells cultured with DMSO (vehicle control) or 0.5 µg/mL chelidonine for 12 hours. The framed subset represents dead cells, including both apoptotic and necrotic subpopulations. Color reflects event density, with warmer colors representing regions containing more cells. (B) Bar chart showing the fraction of dead cells (apoptotic and necrotic) after 24 hours of treatment with DMSO (vehicle control) or the indicated concentrations of chelidonine. (C) Bar chart showing the fraction of dead cells (apoptotic and necrotic) after 6 hours (light gray bars), 12 hours (dark gray bars), and 24 hours (blue bars) of treatment with DMSO (vehicle control) or the indicated concentrations of chelidonine. Data represent mean ± SD from three independent experiments. **p<0.01, ****p<0.0001, ns: not significant. The percentage of dead cells remained unchanged in DMSO-treated controls throughout the experimental time course. For clarity, statistical comparisons for control cells are not shown in the figure. Kit225 K6 cells were cultured with chelidonine or DMSO alone for the above-mentioned durations in the presence of 200 units/mL IL-2. Cell were then stained with Alexa Fluor 647-conjugated Annexin V and PI and analyzed by flow cytometry (n=100000 cells/sample). (CE: chelidonine, DMSO: dimethyl-sulfoxide, PI: propidium iodide).

SUPPLEMENTARY FIGURES

Supplementary Figure 2.

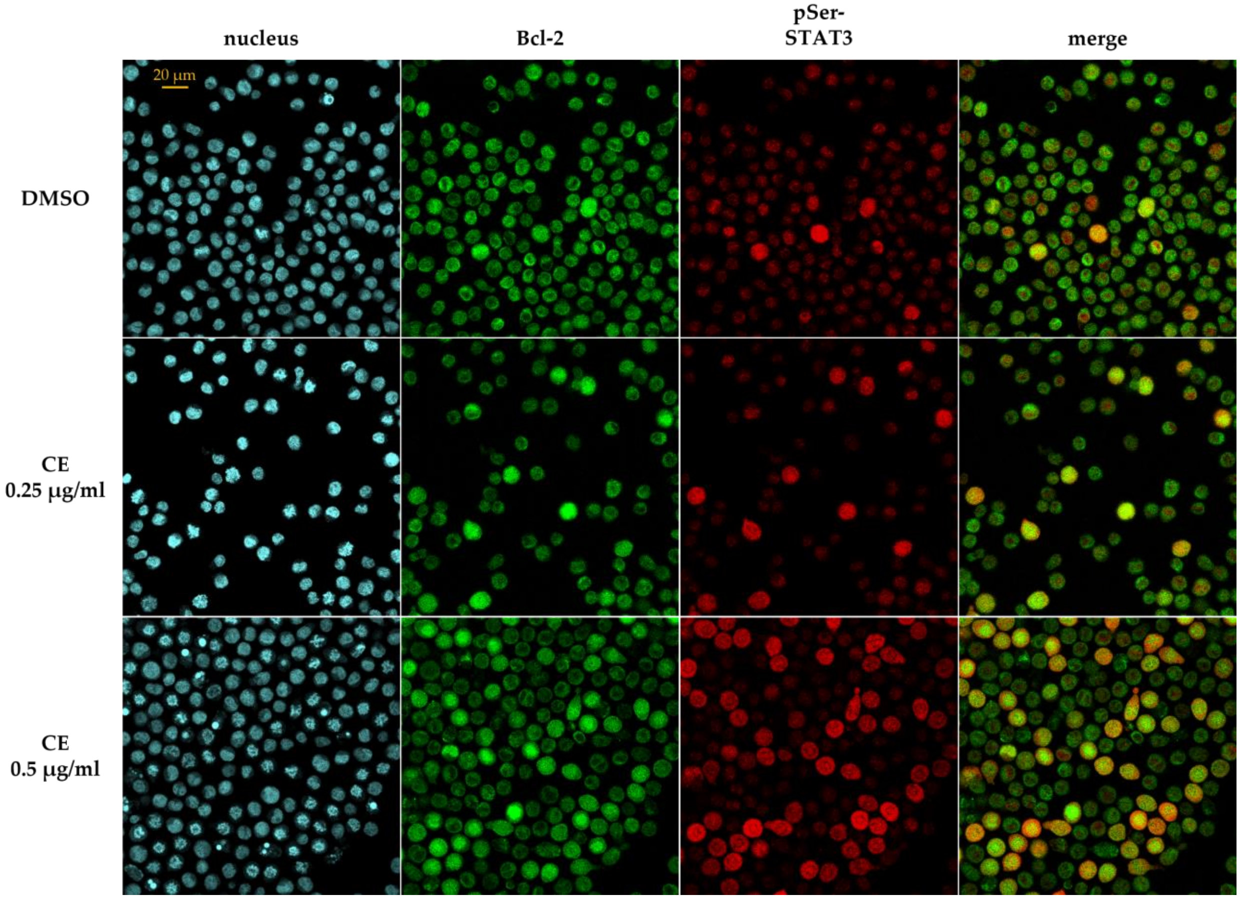

Representative confocal microscopy images showing coordinated changes in pSer-STAT3 and Bcl-2 levels in cells cultured with DMSO (top row), 0.25 μg/mL chelidonine (middle row) and 0.5 μg/mL chelidonine (bottom row) for 12 hours. Blue fluorescence marks nuclei stained with DAPI (first column), green fluorescence indicates Bcl-2 detected with phycoerythrin-conjugated anti-Bcl-2 (second column), and red fluorescence denotes pSer-STAT3 detected with Alexa Fluor 647-conjugated anti-pSer-STAT3 (third column). Overlay images (fourth column) show co-expression of pSer-STAT3 and Bcl-2. Kit225 K6 cells were cultured with chelidonine or DMSO alone in the presence of 200 units/mL IL-2. Cells were then subjected to immunofluorescence staining and analyzed by confocal microscopy. (CE: chelidonine, DMSO: dimethyl sulfoxide, pSer-STAT3: STAT3 phosphorylated on the serine 727 residue)

SUPPLEMENTARY FIGURES

Supplementary Figure 3.

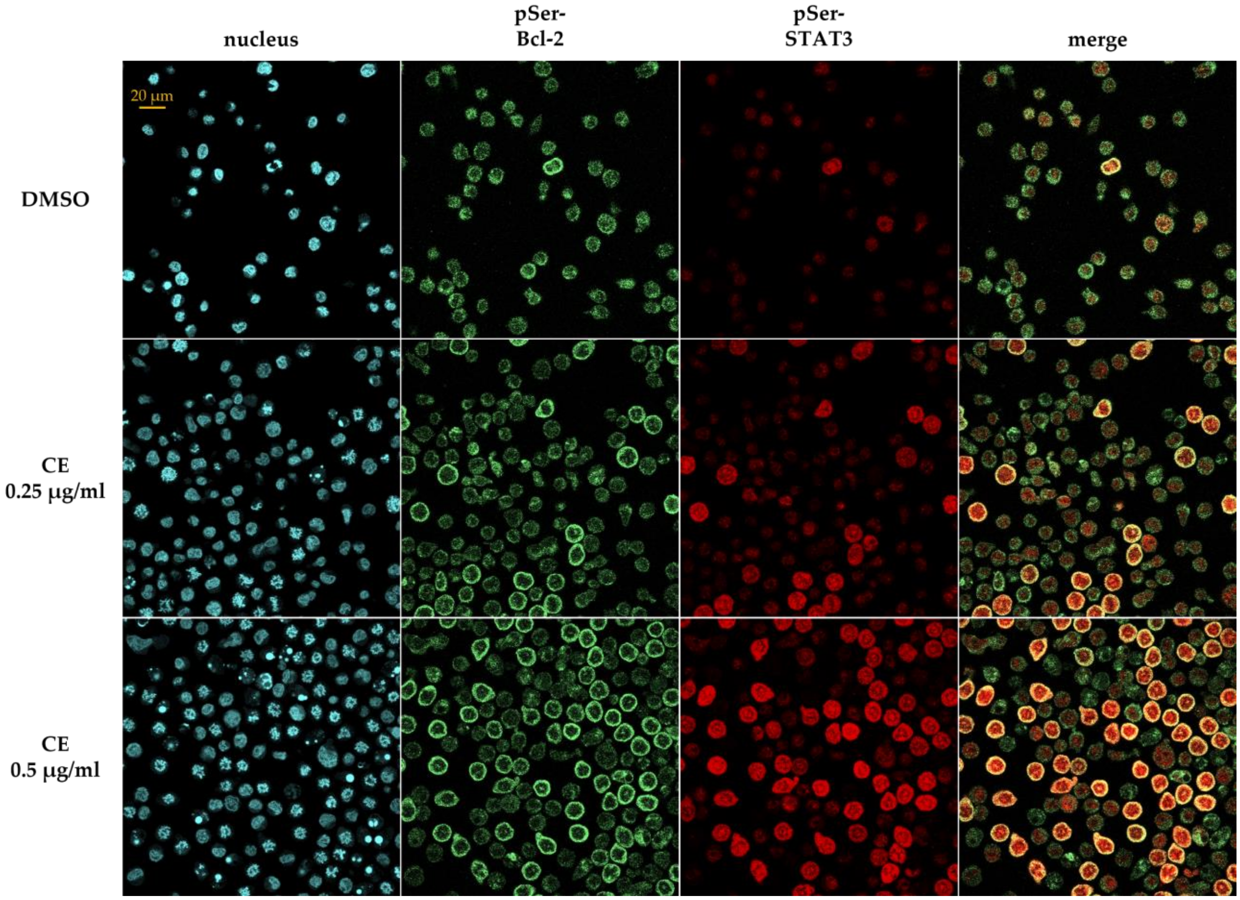

Representative confocal microscopy images showing coordinated changes in pSer-STAT3 and pSer-Bcl-2 levels in cells cultured with DMSO (top row), 0.25 μg/mL chelidonine (middle row) and 0.5 μg/mL chelidonine (bottom row) for 12 hours. Blue fluorescence marks nuclei stained with DAPI (first column), green fluorescence indicates pSer-Bcl-2 detected with Alexa Fluor 488-conjugated anti-pSer-Bcl-2 (second column), and red fluorescence denotes pSer-STAT3 detected with Alexa Fluor 647-conjugated anti-pSer-STAT3 (third column). Overlay images (fourth row) show co-expression of pSer-STAT3 and pSer-Bcl-2. Kit225 K6 cells were cultured with chelidonine or DMSO alone in the presence of 200 units/mL IL-2. Cells were then subjected to immunofluorescence staining and analyzed by confocal microscopy. (CE: chelidonine, DMSO: dimethyl sulfoxide, pSer-Bcl-2: Bcl-2 phosphorylated on the serine 70 residue, pSer-STAT3: STAT3 phosphorylated on the serine 727 residue)

SUPPLEMENTARY FIGURES

Supplementary Figure 4.

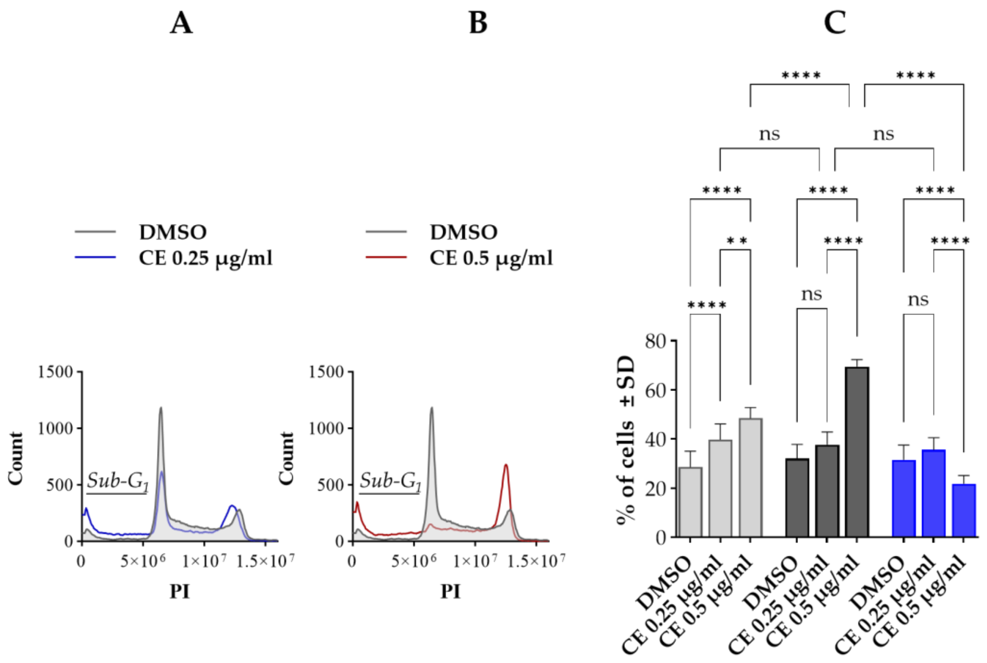

Chelidonine induces G2/M cell cycle arrest in Kit225 K6 cells. (A-B) Representative flow cytometry histograms showing cell cycle distribution based on propidium iodide staining (DNA-content) of cells cultured with DMSO or chelidonine for 12 hours. The gray histogram denotes control cells, while blue and red histograms correspond to 0.25 and 0.5 μg/mL chelidonine treatments, respectively. (C) Bar chart showing the percentage of cells arrested in the G2/M phase after 6-, 12- and 24-hour treatment with chelidonine or DMSO alone (light gray, dark gray and blue bars, respectively). Percentages are expressed as the mean±SD values of three independent experiments. **p<0.01, ****p<0.0001, ns: not significant. The percentage of G2/M cells remained unchanged in DMSO-treated controls throughout the experimental time course. For clarity, statistical comparisons for control cells are not shown in the figure. Kit225 K6 cells were cultured with chelidonine or DMSO alone for the above-mentioned durations in the presence of 30 units/mL IL-2. Cells were then stained with PI and analyzed by flow cytometry (n=100000). (CE: chelidonine, DMSO: dimethyl sulfoxide, PI: propidium iodide).

SUPPLEMENTARY FIGURES

Supplementary Figure 5.

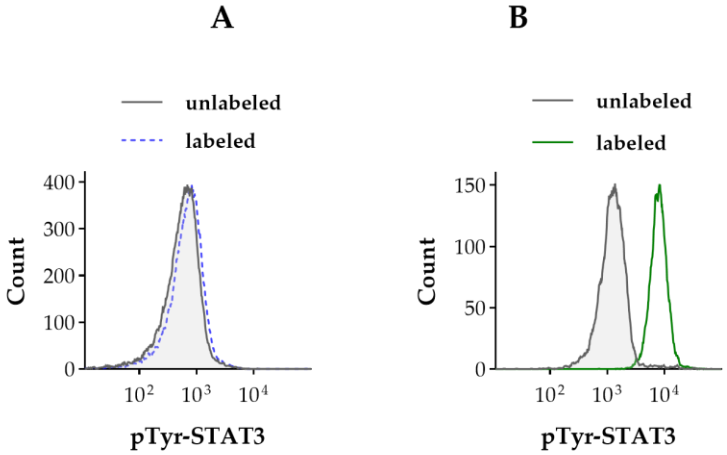

Absence of STAT3 tyrosine phosphorylation in Kit225 K6 cells deprived of IL-2. (A) Representative histogram showing cell-by-cell distribution of basal pTyr-STAT3 level in cells deprived of IL-2 (dashed blue histogram). The gray-filled histogram represents the background (unlabeled cells). (B) Representative histogram showing cell-by-cell distribution of basal pTyr-STAT3 level in cells cultured with IL-2 (green histogram). The gray-filled histogram represents the background (unlabeled cells).
